# Vascular fibrosis and extracellular matrix remodelling in post-COVID 19 conditions

**DOI:** 10.1016/j.imj.2024.100147

**Published:** 2024-10-19

**Authors:** Anna Kamdar, Robert Sykes, Cameron R. Thomson, Kenneth Mangion, Daniel Ang, Michelle AW Lee, Tom Van Agtmael, Colin Berry

**Affiliations:** a School of Cardiovascular and Metabolic Health, University of Glasgow, Glasgow G12 8TA, UK; b West of Scotland Heart and Lung Centre, Golden Jubilee National Hospital, Glasgow G81 4DY, UK; c Department of Cardiology, Queen Elizabeth University Hospital, NHS Greater Glasgow and Clyde Health Board, Glasgow G51 4TF, UK

## Abstract

Causal associations between viral infections and acute myocardial injury are not fully understood, with mechanisms potentially involving direct cardiovascular involvement or systemic inflammation. This review explores plausible mechanisms of vascular fibrosis in patients with post-COVID-19 syndrome, focusing on extracellular matrix remodelling. Despite global attention, significant mechanistic or translational breakthroughs in the management of post-viral syndromes remain limited. No effective pharmacological or non-pharmacological interventions are currently available for patients experiencing persistent symptoms following COVID-19 infection. The substantial expansion of scientific knowledge resulting from collaborative efforts by medical experts, scientists, and government organisations in undertaking COVID-19 research could inform treatment strategies for other post-viral syndromes and respiratory illnesses. There is a critical need for clinical trials to evaluate potential therapeutic candidates, providing evidence to guide treatment decisions for post-COVID-19 syndromes.

## Highlights

• COVID-19 research could be applicable to and inform on other post-viral syndromes and respiratory illnesses.
• Histopathological evidence of increased collagen deposition, abnormal CMR findings and upregulated extracellular matrix remodelling related pathways suggests that systemic, and vascular inflammation, may contribute to symptom burden in post-COVID-19 syndromes.
• Identification of candidate target pathways remains a challenge in myocarditis patients due to the complexity of presentation and lack of prospective tissue collection.
• Trials are needed to provide clinical and scientific evidence to inform treatment decisions in post-COVID-19 syndromes.

## Graphical abstract

Central illustration

**Figure:**
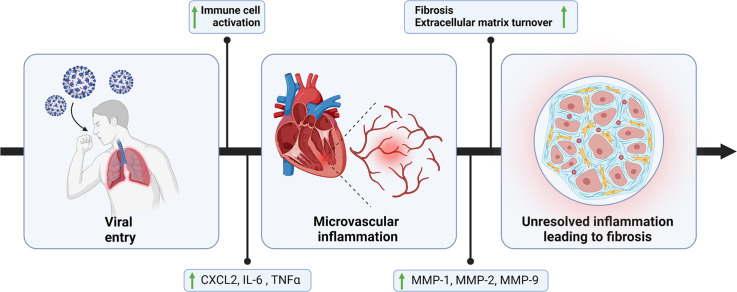

## 1 Introduction

Coronavirus Disease 2019 (COVID-19) represents one of the greatest challenges to healthcare and society in modern times. The acute global increase in unscheduled care and delay of planned procedures and consultations have stretched demands on healthcare systems, further widening healthcare inequalities. During the pandemic, there was a considerable expansion of scientific knowledge, achieved through the combined efforts of medical experts, scientists, and government organisations [1]. As new variants continue to emerge, it will be of critical importance to provide rigorous scientific evidence to guide healthcare policy moving forwards.

Persistent illness following infection with COVID-19 is estimated to affect 145 million people, approximately 3.7% of all COVID-19 cases, and appears to be more prevalent in individuals hospitalized with COVID-19 compared to non-hospitalized individuals (52% vs. 38%) [2,3]. However, initial COVID-19 severity does not appear to correlate with the persisting symptom burden for those not admitted to the hospital. This discrepancy in the literature may be due to the lack of prospective studies in the field.

Two working definitions are currently provided for post-COVID-19 syndrome by the UK National Institute for Clinical Excellence: (1) Ongoing symptomatic COVID-19, encompassing persistent symptoms up to 12 weeks from symptom onset; and (2) post-COVID-19 syndrome, which includes patients with persistent symptoms beyond 12 weeks of symptom onset. However, the aetiology of post-acute COVID-19 syndromes, or ‘Long-COVID,’ remains uncertain, and treatments are lacking.

**Fig. 1:**
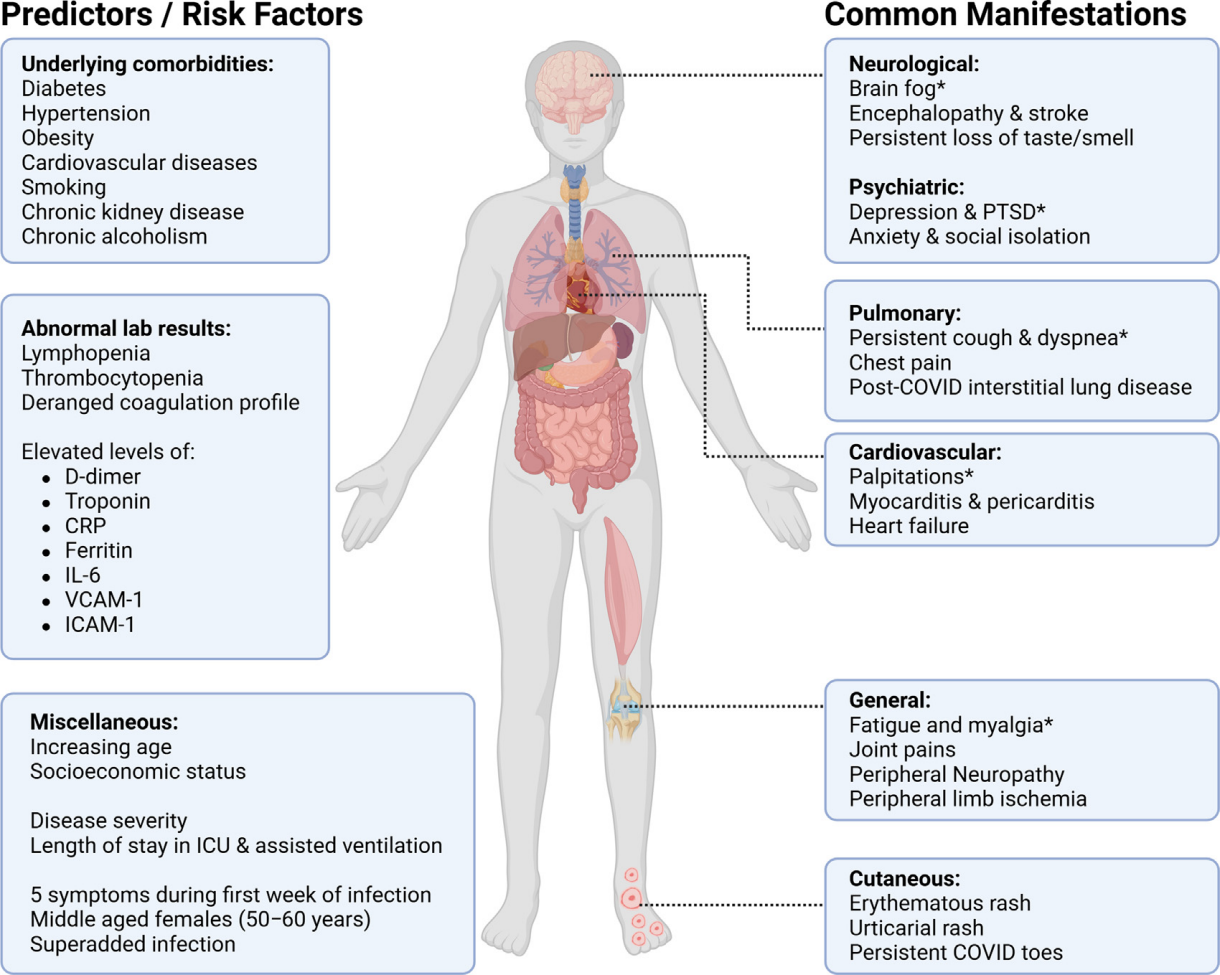
Identified predictors of long-COVID alongside common symptoms and signs. *=hallmark symptom.

Host factors (Fig. 1) identified as contributing to symptom persistence include female sex, increasing age, epigenetics, lifestyle, comorbidity burden, vaccine status, pre-existing cardiovascular disease, and cardiovascular risk factors [[4], [5], [6]]. SARS-CoV-2 specific factors (Fig. 1), including the strain, severity of illness, treatment regimen, complicating sequelae of infection, duration of hospital stay, and rehabilitation, contribute to the likelihood of persisting symptoms beyond 12 weeks [[7], [8], [9], [10], [11]]. Given the context of the COVID-19 pandemic, this proportion of patients with persistent symptoms will continue to present a considerable burden of illness affecting patients, families, and the global economy [12,13].

Post-COVID complications continue to be a prioritised research area given the increasing number of individuals experiencing post-COVID-19 syndrome and the potential generalisability of results to other viral pathogens [14]. In the United Kingdom, ∼1.9 million people have self-reported experiencing symptoms of post-COVID-19 syndrome, with the most common symptom being fatigue [15]. Endothelial damage and systemic inflammation are thought to be key contributors to extra-pulmonary organ dysfunction in patients infected with COVID-19, including acute kidney injury and myocarditis [16,17]. However, the natural course leading to cardiac complications due to COVID-19 infection is poorly understood. Hypotheses for the development of cardiovascular sequelae include systemic inflammation, hypoxaemia, and hypotension, which result in vascular injury. Crucially, whether post-COVID syndromes result from a primary process mediated by viral infection of cells, indirect effects of COVID-19 infection, or perhaps direct injury to the cardiovascular system remains unknown [18].

## 2 Cardiovascular matrix remodelling: mechanisms and pathways

The broad spectrum of illness severity during acute initial infection is contributed to by pulmonary microvascular and endothelial dysfunction, as well as thromboembolic burden. SARS-CoV-2 can spread throughout the ciliated pulmonary epithelium, eventually reaching the highly vascular lung alveoli, contributing to the development of pneumonitis [19]. Direct epithelial disruption, metaplasia, and high proliferation rates of SARS-CoV-2 have been observed in the epithelium of patients with adult respiratory distress syndrome (ARDS) [20]. Additionally, myocardial scarring, which suggests previous myocarditis, can be detected in nearly one-third of hospitalized COVID-19 patients using cardiovascular magnetic resonance (CMR) imaging [[21], [22], [23]].

The Cardiovascular Imaging in COVID-19 study (CISCO-19) [17] is an ongoing prospective cohort study of 159 patients hospitalized for COVID-19 and 29 non-COVID controls matched for age, sex, ethnicity, and morbidity. For the primary endpoint of adjudicated likelihood of myocarditis, it was found that myocarditis was very likely or probable in 54% of these hospitalized participants. Initial reporting also found persisting cardio-renal, vascular, and inflammatory abnormalities, haemostasis pathway activation, and impairments in health status up to one year. These included increased C-reactive protein, intracellular adhesion molecule 1 (ICAM-1), and vascular cell adhesion protein 1 (VCAM-1). In addition, N-terminal prohormone of brain natriuretic peptide (NT-proBNP) and Factor VIII were elevated, reflecting upregulated haemostasis pathway activation [17]. Post-COVID patients also had worse health-related quality of life (EQ5D-5L), illness perception, anxiety, and depression compared to controls, which was associated with adjudicated myocarditis [17]. This inclusion of coronary angiography with FFR_CT_ adds certainty in identifying flow-limiting coronary artery disease, a potential confounder for myocardial inflammation. Moreover, multiorgan imaging was unique to the CISCO-19 study protocol.

In a pre-specified analysis of stress perfusion at 1.5 Tesla (T) or 3.0 T CMR imaging and CT coronary angiography with fractional flow reserve (FFR_CT_) (*n*=102) in the COVID-HEART study, inducible myocardial ischaemia was present in 20% of participants hospitalized with COVID-19 [24]. Microvascular thrombosis patterns of late gadolinium enhancement (LGE) were present, implicating that microcirculatory injury could be a potential mechanism for persistent symptoms. However, compared to age, sex, and cardiovascular risk-factor matched controls, there was no significant difference in the degree of inducible ischaemia. The authors also demonstrated that global hyperaemic myocardial blood flow was a multivariable associate of health-related quality of life (EQ-5D-5L score) and predicted VO_2max_. In addition, evidence of a proinflammatory state was suggested, as biomarkers of haemostasis (antithrombin, protein S, and VCAM-1) were also multivariable associates of VO_2__max_. These findings remained significant when compared to similarly comorbidity-matched COVID-negative controls, suggesting enhanced vascular injury pathways. The analysis of COVID-HEART focussed on imaging and biomarker data, which may not capture the full complexity of post-COVID syndromes. For instance, patients with new or persistent symptoms may have had an increased likelihood of positive CMR findings. Additionally, distinct mechanisms may define subgroups with multi-organ involvement.

Similarly, Yar and colleagues presented work involving 43 controls and 95 post-hospitalized COVID patients [25]. The presence of any LGE was higher in COVID-19 patients (66% vs. 37%, *p*<0.01), as was the presence of LGE suggestive of previous myocarditis (29% vs. 9%, *p* = 0.01). However, this study was not associated with intensive care unit treatment, greater symptomatic burden, or ventricular dysfunction at 9 months follow-up. The study design, however, lacked a formal diagnosis of myocarditis using the Lake-Louise criteria, although CMR results were verified by a level 3 accredited imager. Cardiopulmonary exercise testing was not performed in this population, limiting conclusions about the functional impact of the observed myocardial changes. Since this study was conducted in a post-acute population, it cannot be concluded that myocardial scarring was a direct result of COVID-19 infection.

CMR studies have revealed distinct aetiologies of acute myocardial injury, including myocarditis, microvascular thrombosis, and myocardial infarction in combination with pre-existing fibrosis with a non-ischemic pattern. The prognostic implications of these findings need to be clarified through longitudinal follow-up studies [17], given the limitations of existing studies. Despite these limitations, the detection of myocardial scarring might provide beneficial clinical information to the care team.

## 3 Vascular remodelling and extracellular matrix remodelling

Within tissues, vascular remodelling is a complex process involving an interplay between inflammation, calcification, and collagen deposition. It can occur both as a response to injury and disease, but also as part of the normal ageing process. Studies have highlighted increased inflammatory markers, including interleukin 6 (IL-6), C-X-C motif chemokine ligand 10 (CXCL10), tumour necrosis factor (TNF)-α, interleukin 1β (IL-1β), and Granulocyte-macrophage colony-stimulating factor (GM-CSF), which are associated with the severity of acute illness [[26], [27], [28], [29]]. It is possible that the inflammation seen in acute COVID-19 results in persistent inflammation, potentially due to immune dysregulation. These elevations in inflammatory markers are also paired with enhanced markers of thrombosis and endothelial injury [26], suggesting vascular injury.

**Fig. 2:**
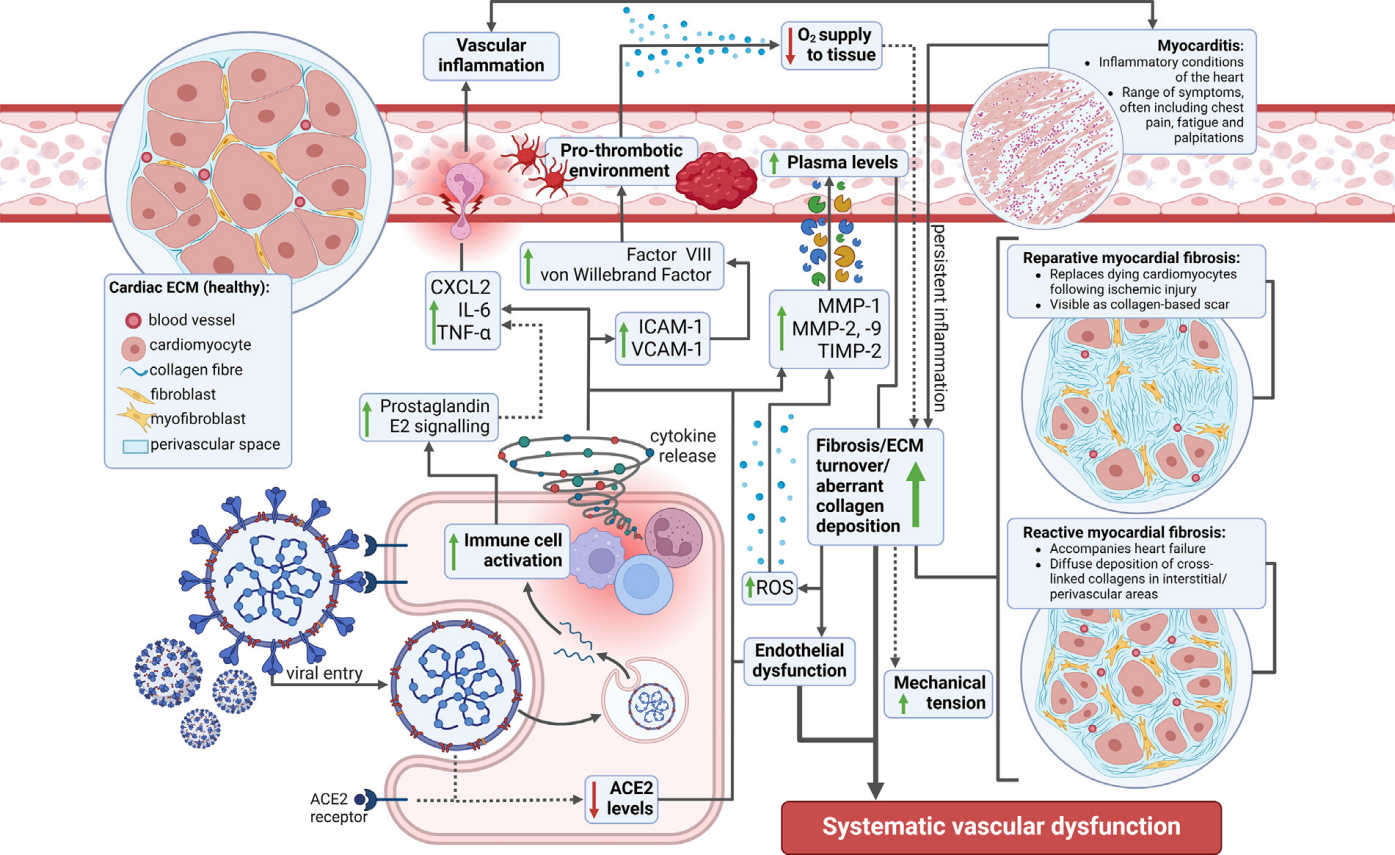
Potential mechanisms of vascular remodelling post-COVID. COVID-19 viral entry is mediated by the angiotensin converting enzyme 2 (ACE2) receptor leading to upregulation of pathways implicated in inflammation, haemostasis activation and extracellular matrix turnover.

Reductions in serum elastase activity and increases in serine protease inhibitor concentration may serve as markers of arterial vessel wall inflammatory changes [[30], [31], [32]]. In addition, microcirculation alterations have been observed in patients requiring mechanical ventilation due to severe COVID-19, increasing endothelial permeability and the likelihood of ARDS [33]. Findings include reduced density of small arteries, damage to the glycocalyx, and increased markers of endothelial activation such as von Willebrand factor-cleaving protease and vascular endothelial growth factor, which were correlated with inpatient mortality in this cohort [33]. Human resistance arteries isolated from hospitalized COVID-19 patients demonstrated increased uptake of aniline blue and picrosirius red compared with hospitalized age- and sex-matched controls. The relationship between systemic tissue inflammation and impairments to quality of life after COVID-19 infection is not well characterised. Possible mechanisms of vascular remodelling post-COVID are illustrated in Fig. 2.

Blood sample comparisons of controls, post-COVID and long-COVID are beginning to reveal the heterogeneity of immune responses between participants to viral immune challenge. Temporal relationships between samples are necessary to ascertain if inflammatory profiles seen in patients are indeed persistent and contributing to fibrotic phenotypes. Serum and peripheral blood mononuclear cell samples from patients have shown upregulation of inflammatory cytokines during the acute infection phase of patients who have developed long COVID when compared to control populations [34,35]. In addition, patients with long COVD have been identified as having higher levels of vascular endothelial growth factor during their acute infection compared to controls [34].

COVID-19 reinfection leads to both acute and long-term endothelial dysfunction, lasting up to 6 months or more. In a study of human cardiac resistance arteries using video microscopy, investigators found persistent endothelial dysfunction months after the initial COVID infection [36] when compared to historical controls, although this study lacked sufficient power. These findings suggest an elevated risk of cardiovascular events and significant endothelial dysfunction for several months following hospitalization for COVID-19. The impact of reinfection on endothelial function, particularly in the vasculature, remains uncertain. In a large-scale national healthcare cohort study based on US Department of Veteran Affairs databases [37], which linked electronic health records, outcomes were compared among patients with first-time infection (*N* = 257,427), reinfection (*N* = 38,926), and a non-infected control group (*N* = 5,396,855). Reinfection significantly increased the risk of cardiovascular sequelae compared to first-time infection, with a hazard ratio (HR) of 2.36 (95% CI: 2.23–2.51). Additionally, reinfection was associated with higher risks of all-cause mortality (HR 2.14; 95% CI: 1.97–2.33) and hospitalization (HR 2.98; 95% CI: 2.83–3.12) at 6 months. Reinfection may be a contributing factor to persisting illness post-COVID; however, the mechanisms underlying this remain unclear.

## 4 ACE2 and vascular remodelling in COVID-19 infection

Angiotensin converting enzyme 2 (ACE2) was identified in 2003 as a functional receptor for severe acute respiratory syndrome-related coronavirus [38]. Unlike other coronaviruses, SARS-CoV-2 does not bind to alternative entry receptors such as aminopeptidase N or dipeptidyl peptidase 4 [39]. ACE2 is ubiquitously expressed, with the highest levels found in the small intestine, testis, kidneys, heart, thyroid, adipose tissue, and lungs [40]. To infect host cells, the coronavirus spike glycoprotein binds with cell surface receptors. Disruption of SARS-CoV-2 spike glycoprotein-ACE2 binding is a potential therapeutic target. Studies in ACE2-deficient murine models have demonstrated the protective role of ACE2 in acute lung injury of non-COVID-19 aetiology [41,42]. ACE2 knockout models also exhibit increased vascular stiffness and injury, likely due to the angiotensin-II effect, which increases oxidative stress, apoptosis, and vascular smooth muscle cell phenotypic switching [[43], [44], [45]]. Additionally, blood proteome studies have highlighted elevated levels of p-selectin and angiotensin-I in post-COVID-19 syndrome when compared to hospitalized COVID-19 patients and healthy controls [46].

The ACE2/Ang-(1-7) pathway is protective against vascular fibrosis by regulating extracellular matrix (ECM) turnover and pro-fibrogenic pathways. Loss of ACE2 promotes the expression of angiotensin II-associated fibrosis genes, including procollagen type I/III and TGF-β [43]. This downregulation of ACE2, coupled with potential increases in angiotensin II, may contribute to inflammation and fibrosis in post-COVID syndromes [42]. The immune system, central to the human viral response, is divided into innate and adaptive responses. The innate immune system is the main defence against pathogens in most organisms [[47], [48]]. Prostaglandins are produced by cells in response to stimuli, including infection. Specifically, prostaglandin E2 (PGE2) plays a role in blood pressure maintenance, vasodilation, and the stimulation of inflammation during host responses to infection [49]. PGE2 signalling cascades are complex and tissue-specific. Prostaglandins can exhibit both proinflammatory and anti-inflammatory properties during the innate immune response. While this variation in PGE2 receptor responses allows for adaptability in cellular infection responses [50], chronic and/or abnormal signalling may contribute to tissue remodelling, as observed in asthma through bronchiole smooth muscle hyperplasia and airway remodelling [51].

In addition, novel spatial transcriptomics have revealed increased expression of myosin light chain kinase and prostaglandin I-synthase [52], alongside upregulation of genes related to ECM regulation and prostaglandin signalling in a case-control study of human resistance arteries. Increased infiltration of fibroblasts and macrophages, along with upregulated ROCK activity, was also observed in COVID-19 arteries [52]. While low levels of prostaglandins enhance immunity, significantly elevated amounts can be deleterious by disrupting lymphocytes [53]. Lymphocyte depletion has been associated with increased in-hospital mortality of COVID-19 patients [54]. Candidate biomarker selection should consider whether immunological findings correlate with quality-of-life outcomes. If these pathways remain upregulated in long-COVID populations, they may provide important mechanistic insights into post-viral matrix remodelling in the vasculature. Understanding tissue-specific mechanisms of vascular dysfunction in both post-COVID and long-COVID population will provide further insight into the immune compartment effects of COVID-19 and potentially other viral infections. Despite global attention, there have been few important mechanistic or translational breakthroughs in the management of post-viral syndromes. Systems-based approaches at cellular, tissue, and circulatory levels are still lacking to address these research questions.

Patients taking immunosuppressive therapies or those with chronic inflammation often experience clinical and subclinical metabolic and cardiovascular comorbidities, increasing the likelihood of poor outcomes following COVID-19 infection [55]. In cases of chronic inflammation, increased oxidative stress may activate matrix metalloproteinases (MMPs) via oxidation of the pro-domain's thiol group, leading to altered ECM turnover [56,57]. Preclinical and translational data from the closely related SARS-CoV-1 may explain why patients with a history of cardiovascular disease are at increased risk of cardiac complications after COVID-19 infection. ACE2 is upregulated in rodents and humans in macrophages, endothelial cells, smooth muscle cells, and myocytes following myocardial infarction [58] which may restore renin-angiotensin-aldosterone system (RAAS) homeostasis in the heart post-MI. Additionally, upregulation of ACE2 protects the heart from adverse cardiac remodelling and dysfunction post-MI [59]. Conversely, downregulation of ACE2 is associated with increased vascular inflammation and plaque burden [45].

Systemic inflammation, direct infection of endothelial cells by SARS-CoV-2, and ACE2 dysfunction have been shown to cause endothelial dysfunction [17,60,61]. The resulting microvascular dysfunction, thrombosis, and occlusion likely contribute to multi-organ dysfunction, particularly in the lungs, heart, and kidneys [17]. COVID-19 patients face a risk of persisting symptoms, which could be explained by coronary and systemic microvascular dysfunction as a unifying mechanism for exertional breathlessness, chest pain, and fatigue [17,60,61] due to underlying subendocardial ischemia. This is mechanistically plausible given the abundance of ACE2 in the endothelial cells of small arteries compared to large conduits, which preferentially express endothelial nitric oxide synthase [62] .

## 5 Matrix metalloproteinases and vascular remodelling in COVID-19 infection

Transmembrane and secreted proteases, as well as their inhibitors, play a key role in matrix remodelling. For an in-depth review on ECM remodelling, we refer the reader to the work by Bonnans et al. [63]. MMPs and their inhibitors, the TIMPS, play a key role in ECM remodelling in both health and disease and are required for the deposition and maintenance of the matrix during development.

MMPs interact with the ECM to remodel proteins such as fibronectin and collagen, among many other targets. They are secreted in an inactive form and require proteolytic cleavage, a process that is tightly controlled by proteases. ECM stability is achieved through a balance of both degradation and secretion of matrix components. Myofibroblast activation following injury enhances matrix production and stimulates fibrotic signalling pathways for repair. MMPs have been identified in pathways including cell proliferation, migration, differentiation, tissue repair, immune regulation, and angiogenesis [[64], [65], [66], [67]].

There are 28 MMPs identified in vertebrates, with 23 of these found in humans, and notably, 14 expressed in the vasculature [68]. These can be classified into collagenases, gelatinases, stromelysins, matrilysins, and membrane-type MMPs [64]. MMPs are secreted with a pro-domain that is cleaved to activate them within the ECM or endoplasmic reticulum, often by other MMPs [69]. Due to their key role in disease and development, MMP gene expression is tightly regulated [70] by transcription factors, which themselves are influenced by extracellular signals such as growth factors, cytokines, and mechanical stress [71]. Another group of ECM-enzymes is the a disintegrin and a metalloprotease domain (ADAMs), which processes and releases mature proteins from membrane-anchored precursors [70]. Metalloproteinase activity, and its inactivation of these, is controlled by a myriad of endogenous inhibitors. Tissue inhibitors of metalloproteinases (TIMPs) are the most prominent and inhibit metalloproteinase activity on a 1:1 stoichiometric ratio [72]. The locality of TIMPs is pivotal to their function, and while TIMPs collectively inhibit all known metalloproteinases, the efficacy of inhibition can vary. For example, TIMP-3 is the only member able to inhibit ADAM-17 [73,74].

To date, studies of serum levels of MMPs in post-COVID patients have suggested temporal relationships. Gelzo and colleagues [75] noted that serum levels of MMP3 were significantly higher in COVID-19 patients compared to controls. This increase in serum levels of MMP3 during COVID-19 infection resolved after one month, suggesting it may be an early event in post-acute sequelae, though the sample size was limited to 12 participants. Additionally, MMP3 levels did not increase further during admission, and baseline MMP3 appeared correlated with World Health Organization staging [75]. Most studies of serum MMPs have focused on the post-acute phase, also highlighting the augmentation of MMP2 and MMP9, independent of the hypertension status of patients [76]. Understanding the temporal and spatial pattern of MMP expression and activation will provide new insights into the matrix remodelling pathways occurring in post-COVID-19 syndromes. Importantly, upregulation of MMPs responsible to matrix turnover is not unique to COVID-19 [77,78] Therefore, findings from COVID-19 studies may be relevant to other viral infections.

## 6 Clinical relevance

Endomyocardial biopsy (EMB) remains the gold standard for diagnosing myocarditis or myocardial fibrosis. However, it is an invasive procedure associated with significant risk of complications [79]. Additionally, there can be low diagnostic yield, especially in cases of patchy or isolated disease [80]. The improvement in sampling devices and uptake in the use of microarrays has helped improve the safety and yield of EMB. In a large study of 222 patients with EMB-proven viral myocarditis, there was an increased mortality of up to 19.2% over a 5-year period [81]. Importantly, the study authors also found the presence of LGE to be the best independent predictor of all-cause mortality and cardiac mortality in this study population, highlighting the clinical utility of CMR in viral myocarditis. The mechanisms contributing to viral myocarditis are complex, and therapeutic options for this specific patient population are limited [82].

As access to CMR improves, it is increasingly being used for assessing persistent inflammation and fibrosis in patients with post-COVID-19 syndromes [23]. The modified Lake Louise Criteria is a consensus statement of CMR findings representative of nonischaemic myocardial inflammation. Myocarditis on CMR is characterized by (1) oedema, (2) hyperaemia, and (3) necrosis or scar [83], which can be derived from the assessment of T1-weighted, T2-weighted, early and late- gadolinium enhancement sequences. In a cohort study of 100 patients by Puntmann et al. [23] a high prevalence (78%) of CMR abnormalities were demonstrated, including elevated native T1 values in 73% of these patients and the presence of LGE in 32%. Huang et al. [84] also observed that 31% of recovered COVID-19 patients had CMR findings suggestive of myocardial fibrosis and elevated extracellular volume on CMR. Patients with increased native T1 and T2 relaxation times likely have ongoing inflammatory processes, while those with normal T2 are more likely to have residual and diffuse myocardial damage. It is recognised that this myocardial damage may not be specific to viral infection and could result from cardiac comorbidities, such as hypertension or cardiomyopathy, which also lead to diffuse fibrosis.

The prevalence of nonischaemic myocardial fibrosis prior to the COVID-19 pandemic in the general population was approximately 7.9% [85]. During the convalescent phase of COVID-19 infection, up to 30% of patients show evidence of nonischemic myocardial fibrosis, far exceeding the expected prevalence in the normal adult population. A history of myocarditis following viral infection might explain persistent symptoms in post-COVID populations [85]. In addition, nonischaemic patterns of myocardial LGE are predominantly seen in patients with acute and healed myocarditis, findings strongly linked to adverse outcomes [81,86,87] including all-cause mortality.

Post-mortem assessment of COVID-19 and Influenza A patients have confirmed the presence of secondary lobular microischemia and prolonged blood vessel neoformation through intussusceptive angiogenesis [88]. This microvascular pathology may not be detectable with current standard-of-care clinical imaging technologies. Invasive coronary microvascular function testing in patients following COVID-19 infection has demonstrated abnormal coronary flow reserve and a propensity towards microvascular spasm. The most frequent finding was a combination of impaired endothelial-dependent and -independent relaxation [89,90]. It is plausible that ischemia with non-obstructive myocardial ischemia (INOCA) is prevalent in post-COVID-19 patients with new-onset chest pain, though the exact aetiology remains unknown. Stratified treatment of this patient cohort following the diagnosis of INOCA has improved quality of life, as measured by the change in Seattle Angina Questionnaire Score [89] consistent with results from other stratified INOCA studies [91]. The main mechanism underlying coronary artery spasm is thought to involve increased ROCK myosin light chain phosphorylation, leading to vascular smooth muscle cell hypercontraction [92]. Considering findings in post-COVID resistance arteries [52] it is plausible that there are shared mechanisms.

## 7 Therapeutic considerations

Cardiac fibrosis remains a significant issue in cardiovascular disease management. Vascular fibrosis is progressive, exacerbating arterial stiffness and extending into the surrounding interstitial space. Fibrosis affects both large and small arteries; in larger vessels, this stiffening can lead to hemodynamic damage to peripheral tissues. Myofibroblasts play a critical role in this remodelling process, exhibiting both proliferative and secretory characteristics that contribute to ECM turnover, collagen deposition, fibrotic scarring, and subsequent cardiac dysfunction [93]. Therapeutic development is challenging due to the complex mechanisms underlying fibrosis and the lack of specific targets on myofibroblasts.

Current clinical trials in cardiac fibrosis have shown modest results. The most promising data comes from treatments involving RAAS inhibitors. Several clinical studies [[94], [95], [96], [97]] have shown that both ACE inhibitors and angiotensin receptor blockers reduce cardiac fibrosis in patients, independent of their antihypertensive effects. However, these studies have small sample sizes and lack sufficient statistical power to determine the effect of myocardial fibrosis/scarring on hard clinical endpoints or quality-of-life outcome measures. Large, long-term clinical studies are needed to definitively test these effects. The modest efficacy of these treatments highlights the challenges of translating research findings from animal models to humans.

Recent advancements have introduced chimeric T cell receptor (CAR-T) therapies into cardiovascular research. Aghajanian et al. identified fibroblast activation protein (FAP), which is overexpressed on activated myofibroblasts compared to quiescent cardiac fibroblasts [98]. They engineered CAR-T cells to target FAP, which significantly reduced cardiac fibrosis in injured mice, showcasing the potential of CAR-T therapy. Nevertheless, further research is necessary to optimize this approach, as variations in CAR-T engineering can significantly affect targeting affinities and anti-fibrotic effects. Identification of specific gene signatures in healthy versus diseased states will be required [99]. Moreover, manufacturing remains a large barrier to development, as many studies use autologous or allogenic T cells. The manufacturing time of autologous products limits their use in the acute period of myocarditis, but they may still have utility in the acute period of myocarditis. The use of allogenic T cells carries the risk of graft-versus-host disease, though this is being circumvented in cancer therapies [100,101] with the aim to create universal CAR-T therapies. Developing allogenic CAR-T therapies with a reduced to negligible risk of graft-versus-host disease will be essential for their widespread implementation in any disease area.

Rho-kinase (ROCK) activity increases in response to transforming growth factor-β (TGF-β) stimulation [102], potentially mediating the expression of pro-fibrotic genes. TGF-β/Smad inhibitors can downregulate RhoA and ROCK-1 expression, indicating possible crosstalk between the two pathways [103]. Moreover, well-characterized ROCK inhibitors suppress Smad2 expression and TGF-β-mediated synthesis of type I collagen mRNA in both fibroblasts and epithelial cells [103]. The regulation of the actin cytoskeleton via the Rho–Rho kinase axis positions it as a promising therapeutic target for improving vascular function. Research on ROCK has greatly enhanced our understanding of the pro-fibrotic cellular mechanisms underlying cardiovascular disease and post-COVID-19 sequelae [52]. Future clinical trials on the use of ROCK inhibitors to decrease cardiac fibrosis and/or improve vascular remodelling are necessary to fully evaluate ROCK as a therapeutic target.

## 8 Conclusion

The presence of histopathological evidence of increased collagen deposition, abnormal CMR findings, and evidence of upregulated ECM related pathways suggests that systemic and vascular inflammation are contributing factors to symptom burden in post-COVID-19 clinical syndromes. Patient stratification to assess potential benefit to therapeutic interventions within trial designs is encouraged, utilising quantifiable assessments of vascular function, aerobic capacity, and residual inflammation to guide eligibility and maximise potential benefit. Future studies utilising tissue samples alongside clinical data, underpinned by translational research methods, should be prioritised to answer clinically relevant questions that will improve the management of patients with post-COVID-19 syndrome and potentially other viral respiratory illnesses. Patients with myocarditis remain a clinical challenge due to the difficulty in identifying culprit pathways during complex systemic illness. Many current treatments focus on supportive therapy. Therefore, managing these patients requires individualised supportive therapy while identifying potential novel therapeutic candidates. The expansion of scientific knowledge achieved through the combined efforts of medical experts, scientists, and government organisations in COVID-19 research could be applicable to other post-viral syndromes and respiratory illnesses.

## Funding

Ms Anna Kamdar is supported by research funding from the British Heart Foundation MBPhD (FS/MBPhD/22/28011). Robert Sykes is supported by funding from the British Heart Foundation Centre of Research Excellence award (RE/18/6/34217) and NHS Greater Glasgow and Clyde Endowment Funding (GN21CA394). Cameron Thomson is supported by research funding from The University of Glasgow Doctoral Training Programme. Dr Kenneth Mangion is supported by research funding from the Chief Scientist Office (COV/LTE/20/10, COV/GLA/20/05). Ms Michelle Lee is supported by research funding from the British Heart Foundation Centre of Research Excellence (RE/18/6/34217). Professor Tom Van Agtmael is supported by research funding from EPSRC-Horizon Europe (EP/X031721/1 [EU Ref:101072766]) and MRC (MR/R005567/1). Professor Colin Berry is supported by research funding from the British Heart Foundation (FS/17/26/32744; PG/18/52-33892; RE/18/6/34217) and Medical Research Council (MR/SO18905/1).

## CRediT authorship contribution statement

**Anna Kamdar:** Conceptualization, Investigation, Methodology, Project administration, Writing – original draft, Writing – review & editing. **Robert Sykes:** Conceptualization, Investigation, Methodology, Writing – original draft, Writing – review & editing. **Cameron R. Thomson:** Conceptualization, Data curation, Methodology, Visualization, Writing – original draft, Writing – review & editing. **Kenneth Mangion:** Conceptualization, Data curation, Investigation, Supervision, Writing – review & editing. **Daniel Ang:** Methodology, Project administration, Writing – original draft, Writing – review & editing. **Michelle AW Lee:** Conceptualization, Investigation, Writing – original draft, Writing – review & editing. **Tom Van Agtmael:** Conceptualization, Supervision, Writing – original draft, Writing – review & editing. **Colin Berry:** Conceptualization, Project administration, Supervision, Writing – original draft, Writing – review & editing.

## References

[1] ‘COVID-19 response | United Nations’. Accessed December 12, 2022. Available: https://www.un.org/en/coronavirus

[2] Wulf Hanson S., Abbafati C., Aerts J.G. (2022). A global systematic analysis of the occurrence, severity, and recovery pattern of long COVID in 2020 and 2021. medRxiv.

[3] Pérez-González A., Araújo-Ameijeiras A., Fernández-Villar A. (2022). Long COVID in hospitalized and non-hospitalized patients in a large cohort in Northwest Spain, a prospective cohort study. Sci Rep.

[4] Lazaros G., Oikonomou E., Theofilis P. (2020). The impact of COVID-19 pandemic on adult cardiac surgery procedures. Hellenic J Cardiol.

[5] Vassilikos V.P., Pagourelias E.D., Katsos K. (2020). Impact of social containment measures on cardiovascular admissions and sudden cardiac death rates during Coronavirus Disease (COVID-19) outbreak in Greece. Hellenic J Cardiol.

[6] Oikonomou E., Aznaouridis K., Barbetseas J. (2020). Hospital attendance and admission trends for cardiac diseases during the COVID-19 outbreak and lockdown in Greece. Public Health.

[7] Antonelli M., Pujol J.C., Spector T.D. (2022). Risk of long COVID associated with delta versus omicron variants of SARS-CoV-2. Lancet.

[8] Sugiyama A., Miwata K., Kitahara Y. (2022). Long COVID occurrence in COVID-19 survivors. Sci Rep.

[9] Thompson E.J., Williams D.M., Walker A.J. (2022). Long COVID burden and risk factors in 10 UK longitudinal studies and electronic health records. Nat Commun.

[10] Bai F., Tomasoni D., Falcinella C. (2022). Female gender is associated with long COVID syndrome: a prospective cohort study. Clin Microbiol Infect.

[11] Chlamydas S, Papavassiliou AG, Piperi C (2021 Mar). Epigenetic mechanisms regulating COVID-19 infection. Epigenetics.

[12] Richards F., Kodjamanova P., Chen X. (2022). Economic burden of COVID-19: a systematic review. Clinicoecon Outcomes Res.

[13] NIHR, ‘Living with Covid19 – second review’, 2021, doi: 10.3310/THEMEDREVIEW_45225.

[14] ‘Developing a global research agenda for public health and social measures: research priorities for COVID-19′. Accessed January 10, 2024. Available: https://www.who.int/news/item/05-06-2023-developing-a-global-research-agenda-for-public-health-and-social-measures-research-priorities-for-covid-19

[15] ‘Coronavirus (COVID-19) latest insights - office for national statistics’. Accessed December 12, 2022. Available: https://www.ons.gov.uk/peoplepopulationandcommunity/healthandsocialcare/conditionsanddiseases/articles/coronaviruscovid19latestinsights/infections#long-covid

[16] Gupta A., Madhavan M.V., Sehgal K. (2020). Extrapulmonary manifestations of COVID-19. Nature Medicine.

[17] Morrow A.J., Sykes R., McIntosh A. (2022). A multisystem, cardio-renal investigation of post-COVID-19 illness. Nat Med.

[18] Ball S., Bannerjee A., Berry C. (2020). Monitoring indirect impact of COVID-19 pandemic on services for cardiovascular diseases in the UK. Heart.

[19] Kuba K., Imai Y., Penninger J.M. (2006). Angiotensin-converting enzyme 2 in lung diseases. Curr Opin Pharmacol.

[20] Duarte-Neto A.N., Monteiro R.A.A, da Silva L.F.F (2020). Pulmonary and systemic involvement in COVID-19 patients assessed with ultrasound-guided minimally invasive autopsy. Histopathology.

[21] Ammirati E., Lupi L., Palazzini M. (2022). Prevalence, characteristics, and outcomes of COVID-19-associated acute myocarditis. Circulation.

[22] Kotecha T., Knight D.S., Razvi Y. (2021). Patterns of myocardial injury in recovered troponin-positive COVID-19 patients assessed by cardiovascular magnetic resonance. Eur Heart J.

[23] Puntmann V.O., Careri M.L., Wieters I. (2020). Outcomes of cardiovascular magnetic resonance imaging in patients recently recovered from coronavirus disease 2019 (COVID-19). JAMA Cardiol.

[24] Artico J., Shiwani H., Moon J.C. (2023). Myocardial involvement after hospitalization for COVID-19 complicated by troponin elevation: a prospective, multicenter, observational study. Circulation.

[25] Yar A. (2023). Cardiac magnetic resonance -detected myocardial injury is not associated with long-term symptoms in patients hospitalized due to COVID-19. PLoS One.

[26] R. S. Thwaites, A. Sanchez Sevilla Uruchurtu, M. K. Siggins, ISARIC4C investigators. Inflammatory profiles across the spectrum of disease reveal a distinct role for GM-CSF in severe COVID-19. Sci Immunol. 2021 Mar 10;6(57):eabg9873. doi:10.1126/sciimmunol.abg9873. 10.1126/sciimmunol.abg9873 PMC8128298 33692097

[27] Filbin M.R., Mehta A., Schneider A.M. (2021). Longitudinal proteomic analysis of severe COVID-19 reveals survival-associated signatures, tissue-specific cell death, and cell-cell interactions. Cell Rep Med.

[28] C.E. Brightling, ‘Clinical characteristics with inflammation profiling of long COVID and association with 1-year recovery following hospitalisation in the UK: a prospective observational study’, 2022, doi:10.1016/S2213-2600(22)00127-8. 10.1016/S2213-2600(22)00127-8 PMC9034855 35472304

[29] Huang C., Wang Y., Li X. (2020). Clinical features of patients infected with 2019 novel coronavirus in Wuhan, China. The Lancet.

[30] Barolet A.W., Nili N., Cheema A. (2001). Arterial elastase activity after balloon angioplasty and effects of elafin, an elastase inhibitor, Arterioscler Thromb Vasc. Biol.

[31] AAssar O.S., Fujiwara N.H., Marx W.F. (2003). Aneurysm growth, elastinolysis, and attempted doxycycline inhibition of elastase-induced aneurysms in rabbits. J Vasc Interv Radiol.

[32] Paczek L., Michalska W., Bartlomiejczyk I. (2008). Trypsin, elastase, plasmin and MMP-9 activity in the serum during the human ageing process. Age Ageing.

[33] Rovas A., Osiaevi I., Buscher K. (2021). Microvascular dysfunction in COVID-19: the MYSTIC study. Angiogenesis.

[34] Patterson B.K., Guevara-Coto J., Yogendra R. (2021). Immune-based prediction of COVID-19 severity and chronicity decoded using machine learning. Front Immunol.

[35] Queiroz M.A.F., das Neves P.F.M., Lima S.S. (2022). Cytokine profiles associated with acute COVID-19 and long COVID-19 syndrome. Front Cell Infect Microbiol.

[36] Y. Nishijima, S.N. Hader, A.J. Hanson et al., ‘Prolonged endothelial-dysfunction in human arterioles following infection with SARS-CoV-2′, doi:10.1093/cvr/cvab339. 10.1093/cvr/cvab339 PMC8689948 34755839

[37] Bowe B., Xie Y., Al-Aly Z. (2022). Acute and postacute sequelae associated with SARS-CoV-2 reinfection. Nature Medicine.

[38] Li W., Moore M.J., Vasllieva N. (2003). Angiotensin-converting enzyme 2 is a functional receptor for the SARS coronavirus. Nature.

[39] Zhou P., Lou Yang X., Wang X.G. (2020). A pneumonia outbreak associated with a new coronavirus of probable bat origin. Nature.

[40] M.Y. Li, L. Li, Y. Zhang, et al., Expression of the SARS-CoV-2 cell receptor gene ACE2 in a wide variety of human tissues, Infect Dis Poverty 9 (1) (2020) 45, doi:10.1186/S40249-020-00662-X. 10.1186/s40249-020-00662-x PMC7186534 32345362

[41] Hoffmann M., Kleine-Weber H., Schroeder S. (2020). SARS-CoV-2 Cell Entry depends on ACE2 and TMPRSS2 and is blocked by a clinically proven protease inhibitor. Cell.

[42] Zambelli V., Bellani G., Borsa R. (2015). Angiotensin-(1-7) improves oxygenation, while reducing cellular infiltrate and fibrosis in experimental acute respiratory distress syndrome. Intensive Care Med Exp.

[43] Zhang Z.Z., Chen L.J., Zhong J.C. (2014). ACE2/Ang-(1-7) signaling and vascular remodeling. Sci China Life Sci.

[44] R.A. Fraga-Silva, F.P. Costa-Fraga , T.M. Murca , et al., Angiotensin-converting enzyme 2 activation improves endothelial function, Hypertension 61 (6) (2013) 1233, doi:10.1161/HYPERTENSIONAHA.111.00627. 10.1161/HYPERTENSIONAHA.111.00627 PMC3733257 23608648

[45] Thomas M.C., Pickering R.J., Tsorotes D. (2010). Genetic Ace2 deficiency accentuates vascular inflammation and atherosclerosis in the ApoE knockout mouse. Circ Res.

[46] Patel M.A., Knauer M.J., Nicholson M. (2022). Elevated vascular transformation blood biomarkers in Long-COVID indicate angiogenesis as a key pathophysiological mechanism. Mol Med.

[47] Litman G.W., Cannon J.P., Dishaw L.J. (2005). Reconstructing immune phylogeny: new perspectives. Nat Rev Immunol.

[48] Sander W.J., O'Neill H.G., Pohl C.H. (2017). Prostaglandin E2 as a modulator of viral infections. Front Physiol.

[49] P. Davies, P.J. Bailey, M.M. Goldenberg, et al. ‘The role of arachidonic acid oxygenation products in pain and inflammation’,vol. 2, pp. 335–357, 2003, doi:10.1146/annurev.iy.02.040184.002003. 10.1146/annurev.iy.02.040184.002003 6100476

[50] Hata A.N., Breyer R.M. (2004). Pharmacology and signaling of prostaglandin receptors: multiple roles in inflammation and immune modulation. Pharmacol Ther.

[51] Vignola A.M., Mirabella F., Costanzo G. (2003). Airway Remodeling in Asthma. Chest.

[52] Sykes R.A., Neves K.B., Alves-Lopes R. (2023). Vascular mechanisms of post-COVID-19 conditions: rho-kinase is a novel target for therapy. Eur Heart J Cardiovasc Pharmacother.

[53] M. Ricke-Hoch , E. Steiiling , L. Lasswitz ,et al., Impaired immune response mediated by prostaglandin E2 promotes severe COVID-19 disease, PLoS One 16 (8) (2021) e0255335, doi:10.1371/JOURNAL.PONE.0255335. 10.1371/journal.pone.0255335 PMC8336874 34347801

[54] Li D., Chen Y., Liu H. (2020). Immune dysfunction leads to mortality and organ injury in patients with COVID-19 in China: insights from ERS-COVID-19 study, Signal Transduct Target. Ther.

[55] Shoenfeld Y., Gerli R., Doria A. (2005). Accelerated atherosclerosis in autoimmune rheumatic diseases. Circulation.

[56] Weiss S.J., Peppin G., Ortiz X. (1985). Oxidative autoactivation of latent collagenase by human neutrophils. Science (1979).

[57] Peppin G.J., Weiss S.J. (1986). Activation of the endogenous metalloproteinase, gelatinase, by triggered human neutrophils. Proceed Nat Acad Sci.

[58] Burrell L.M., Risvanis J., Kubota E. (2005). Myocardial infarction increases ACE2 expression in rat and humans. Eur Heart J.

[59] Zhao Y.X., Yin H.Q., Yu Q.T. (2010). ACE2 overexpression ameliorates left ventricular remodeling and dysfunction in a rat model of myocardial infarction. Hum Gene Ther.

[60] Guzik T.J., Mohiddin A.A., Dimarco A. (2020). COVID-19 and the cardiovascular system: implications for risk assessment, diagnosis, and treatment options. Cardiovasc Res.

[61] Libby P., Lüscher T. (2020). COVID-19 is, in the end, an endothelial disease. Eur Heart J.

[62] Shu X., Keller T.C.S., Begandt D. (2015). Endothelial nitric oxide synthase in the microcirculation. Cell Mol Life Sci.

[63] Bonnans C., Chou J., Werb Z. (2014). Remodelling the extracellular matrix in development and disease. Nat Rev Mol Cell Biol.

[64] Cui N., Hu M., Khalil R.A. (2017). Biochemical and biological attributes of matrix metalloproteinases. Prog Mol Biol Transl Sci.

[65] Liu J., Khalil R.A. (2017). Matrix metalloproteinase inhibitors as investigational and therapeutic tools in unrestrained tissue remodeling and pathological disorders. Prog Mol Biol Transl Sci.

[66] Visse R., Nagase H. (2003). Matrix metalloproteinases and tissue inhibitors of metalloproteinases: Structure, function, and biochemistry. Circ Res.

[67] Fischer T., Senn N., Riedl R. (2019). Design and structural evolution of matrix metalloproteinase inhibitors. Chemistry.

[68] Kass D., Rabinovitch M., Visse R. (2003). Matrix metalloproteinases and tissue inhibitors of metalloproteinases. Circ Res.

[69] Javaid M.A., Abdallah M.N., Ahmed A.S. (2013). Matrix metalloproteinases and their pathological upregulation in multiple sclerosis: an overview. Acta Neurol Belg.

[70] Yong V.W. (2005). Metalloproteinases: mediators of pathology and regeneration in the CNS. Nat Rev Neurosci.

[71] Yan C., Boyd D.D. (2007). Regulation of matrix metalloproteinase gene expression. J Cell Physiol.

[72] Yong V.W., Power C., Edwards D.R. (2001). Metalloproteinases in biology and pathology of the nervous system. Nat Rev Neurosci.

[73] Khokha R., Murthy A., Weiss A. (2013). Metalloproteinases and their natural inhibitors in inflammation and immunity. Nat Rev Immunol.

[74] Murphy G., Knäuper V., Lee M.H. (2003). Role of TIMPs (tissue inhibitors of metalloproteinases) in pericellular proteolysis: the specificity is in the detail. Biochem Soc Symposia.

[75] Gelzo M., Cacciapuoti S., Pinchera B. (2022). Matrix metalloproteinases (MMP) 3 and 9 as biomarkers of severity in COVID-19 patients. Scient Rep.

[76] D`Avila-Mesquita C., Couto A.E.S., Campos L.C.B. (2021). MMP-2 and MMP-9 levels in plasma are altered and associated with mortality in COVID-19 patients. Biomed Pharmacother.

[77] Sellner J., Simon F., Meyding-Lamade U. (2006). Herpes-simplex virus encephalitis is characterized by an early MMP-9 increase and collagen type IV degradation. Brain Res.

[78] Singh B., Fleury C., Jalalvand F. (2012). Human pathogens utilize host extracellular matrix proteins laminin and collagen for adhesion and invasion of the host. FEMS Microbiol Rev.

[79] Kiamanesh O., Toma M. (2021). The state of the heart biopsy: a clinical review. CJC Open.

[80] Bennett M.K., Gilotra N.A., Harrington C. (2013). Evaluation of the role of endomyocardial biopsy in 851 patients with unexplained heart failure from 2000-2009. Circ Heart Fail.

[81] Grn S., Schumm J., Greulich S. (2012). Long-term follow-up of biopsy-proven viral myocarditis: predictors of mortality and incomplete recovery. J Am Coll Cardiol.

[82] Fung G., Luo H., Qiu Y. (2016). Myocarditis. Circ Res.

[83] Ferreira V.M., Schulz-Menger J., Holmvang G. (2018). Cardiovascular magnetic resonance in nonischemic myocardial inflammation: expert recommendations. J Am Coll Cardiol.

[84] Huang L., Zhao P., Tang D. (2020). Cardiac involvement in patients recovered from COVID-2019 identified using magnetic resonance imaging. JACC Cardiovasc Imaging.

[85] Halfmann M.C., Leutkens J.A., Langenbach I.L. (2023). Cardiac MRI findings in patients clinically referred for evaluation of post-acute sequelae of SARS-CoV-2 infection. Diagnostics.

[86] Puntmann V.O., Carr-White G., Jabbour A. (2016). T1-mapping and outcome in nonischemic cardiomyopathy: all-cause mortality and heart failure. JACC Cardiovasc Imaging.

[87] Puntmann V.O., Carr-White G., Jabbour A. (2018). Native T1 and ECV of noninfarcted myocardium and outcome in patients with coronary artery disease. J Am Coll Cardiol.

[88] Ackermann M., Kamp J.C., Weirlein C. (2022). The fatal trajectory of pulmonary COVID-19 is driven by lobular ischemia and fibrotic remodelling. EBioMedicine.

[89] Escaned J., Espejo-Paeres C., Jerónimo A. (2024). Myocardial ischemia of nonobstructive origin as a cause of new-onset chest pain in Long-COVID syndrome. JACC Cardiovasc Interv.

[90] C. Espejo , H. Mejia-Renteria , A. Travieso , et al., Myocardial ischaemia of non-obstructive origin as a cause of new onset anginal chest pain in the long COVID syndrome, Eur Heart J 42 (Supplement_1) (2021), doi:10.1093/EURHEARTJ/EHAB724.1078.

[91] Ford T.J., Stanley B., Sidik N. (2020). 1-year outcomes of angina management guided by invasive coronary function testing (CorMicA). JACC Cardiovasc Interv.

[92] Godo S., Takahashi J., Yasuda S. (2021). Role of inflammation in coronary epicardial and microvascular dysfunction. European Cardiology Review.

[93] Querejeta R., López B., González A. (2004). Increased collagen type I synthesis in patients with heart failure of hypertensive origin: relation to myocardial fibrosis. Circulation.

[94] Shimada Y.J., Passeri J.J., Baggish A.L. (2013). Effects of losartan on left ventricular hypertrophy and fibrosis in patients with nonobstructive hypertrophic cardiomyopathy, JACC Heart. Fail.

[95] Deswal A., Richardson P., Bozkurt B. (2011). Results of the randomized aldosterone antagonism in heart failure with preserved ejection fraction trial (RAAM-PEF). J Card Fail.

[96] Kosmala W., Przewlocka-Kosmala M., Szczepanik-Osadnik H. (2011). A randomized study of the beneficial effects of aldosterone antagonism on LV function, structure, and fibrosis markers in metabolic syndrome. JACC Cardiovasc Imaging.

[97] Kawamura M., Ito H., Onuki T. (2010). Candesartan decreases type III procollagen-N-peptide levels and inflammatory marker levels and maintains sinus rhythm in patients with atrial fibrillation. J Cardiovasc Pharmacol.

[98] Aghajanian H., Kimura T., Rurik J.G. (2019). Targeting cardiac fibrosis with engineered T cells. Nature.

[99] Rafiq S., Hackett C.S., Brentjens R.J. (2019). Engineering strategies to overcome the current roadblocks in CAR T cell therapy. Nat Rev Clin Oncol.

[100] Torikai H., Reik A., Liu P.Q. (2012). A foundation for universal T-cell based immunotherapy: T cells engineered to express a CD19-specific chimeric-antigen-receptor and eliminate expression of endogenous TCR. Blood.

[101] Osborn M.J., Webber B.R., Knipping F. (2016). Evaluation of TCR gene editing achieved by TALENs, CRISPR/Cas9, and megaTAL Nucleases. Mol Ther.

[102] Sandbo N., Lau A., Kach J. (2011). Delayed stress fiber formation mediates pulmonary myofibroblast differentiation in response to TGF-β. Am J Physiol Lung Cell Mol Physiol.

[103] Ji H., Tang H., Lin H. (2014). Rho/Rock cross-talks with transforming growth factor-β/Smad pathway participates in lung fibroblast-myofibroblast differentiation. Biomed Rep.

